# Catch per unit effort and some water quality parameters of Lake Kalgwai Jigawa state, Nigeria

**DOI:** 10.1002/fsn3.573

**Published:** 2017-12-19

**Authors:** Shola G. Solomon, Victoria O. Ayuba, Musa A. Tahir, Victor T. Okomoda

**Affiliations:** ^1^ Department of fisheries and Aquaculture University of Agriculture Makurdi Makurdi Nigeria

**Keywords:** catch, Effort, Fishers, Lake Kalgwai, Water quality

## Abstract

This study investigated the catch per unit effort and water quality of Lake Kalgwai Jigawa state, Nigeria for the period of 10 month (July 2012‐June 2013). The man hours, gears used, and fish catches of the Lake was determined by assessing the fishermen operating on three major landing sites of the Lake, namely Marke (Site I), Dingare (Site II), and Kalgwai (Site III). Water samples from the landing sites were collected and analyzed. Concentration of fishers ranged from 41 (April) to 51 (September). The highest number of fishing hour was observed in August (64 hr), whereas the lowest was in March (49 hr). The average catch per fisher per day ranged from 107 kg/day (December) to 144 kg/day (August) during the study period. An average of 25 days was spent fishing in each month. The result also indicated similarities in the water qualities of all the three sampling sites per months. Based on the result gotten it was concluded that lake Kalgwa is not over fished and water quality are within recommended ranges for fish production.

## 1 INTRODUCTION

Fish supply in Africa is in crisis. Per capita consumption in sub‐Saharan Africa is the lowest in all regions and it is the only part of the world where consumption is declining. The main reason for this decline is the leveling off in capture fish production and the ever‐growing population. World Bank (2004) had estimated that fish production must be increase by 27.7% over if a per capita fish supply of 6.6 kg/year is to be maintained in sub‐Saharan Africa by 2015. However, if capture fisheries is to continue to provide the bulk of fish food for Africans sustained efforts would have to be made to support, promote, and protect small‐scale labor‐intensive (both coastal and inland) fisheries. Investments in applied research and capacity building will be required to improve and strengthen the socio‐institutional mechanisms underpinning the fisheries management process. But investments to improve environmental management are also required to sustain fisheries, especially in inland fisheries where increasing pressure on land and water is leading to high environmental degradation. These inland fisheries provide the basis of the livelihoods and therefore the indirect support to food security for millions of people, (World Fish Center, 2005).

The poor and uneconomic management of reservoir and lake fisheries is another major issue of concern. Therefore, creation of dams provides an ecosystem for the proliferation of wide range of aquatic organisms including fish thus promoting socio‐economic activities of the surrounding communities (Fawole, 2002). However, closing fishing areas and regulating the use of fishing gear can result in more profitable catches and higher incomes. For instance, a 12‐year study on fish caught in three locations off the coast of Kenya showed that fishing close to an area with restrictions led to larger catches of fish with higher market value (Spore, 2011). Alternatively, sustainable fishing regulations have been found to facilitate replenishment of stocks. Restricting catches, imposing a minimum size for fishing and halting fishing for a certain period each year are strategies that enable species to regenerates, whereas everyone benefits including fishermen and consumers. Fishermen at Lake Albert, Uganda, have witnessed a rapid change and firsthand benefits of a 20‐month fishing ban imposed between March 2010 and January 2011 (Spore, 2012). Some species of fish that were seldom found overtime became dominate catches of the lake hence, market prices for this fish fell by 40%.

Fishes are generally poikilotherms (cold blooded) and therefore their metabolic rates are strongly influenced by the external environment conditions. The thermal tolerance of fish for instance could be lethal, controlling, and directly influencing the responses of fish (Fry, 1971). In many cases, changes in thermal conditions are also accompanied by changes in other water characteristics such as water levels, changes in composition and amount of food, changes in acidity and other chemical characteristics (Schindler, 2001). Therefore, seasonal influences and instances when such changes occur may be equally or even more severe than changes expressed on an annual basis (Pearson & Dawson, 2003). Fishes as been exploited as excellent bioindicators of water quality changes (Idodo‐Umeh, 2002; Ogbeibu & Victor, 1989; Oguzie, 2003; Yamazaki, Tanizaki, & Shinikawa, 1996). The justification lies in the fact that fish communities respond to episodic events and therefore integrate environmental conditions over time. This study is therefore designed to evaluate the catch per unit effort of Lake Kalgwai for 10 months in relation to the water quality parameters.

## 2 MATERIALS AND METHODS

**Figure 1:**
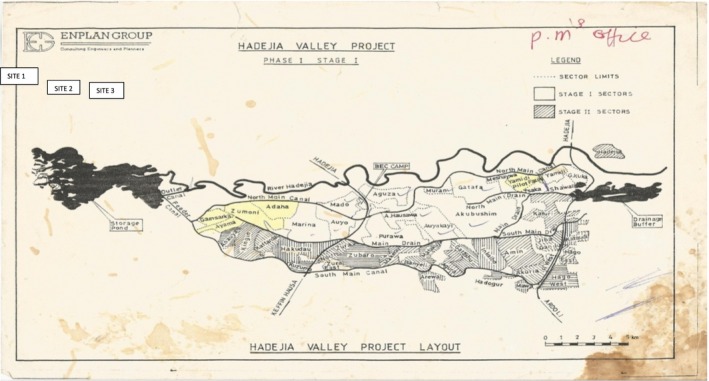
Kalgwai Dam Area Showing the Landing Sites (SOURCE: Hadejia ‐ Jama'are River Basin Development Authority)

Kalgwai Barrage Dam is situated in Auyo Local Government Area of Jigawa State, Nigeria (Figure 1). It was impounded on River Hadeja in 1984 by the Federal Government of Nigeria for the purpose of irrigation under the then HadeJia Valley Irrigation Project which was coordinated by Hadejia‐Jama'are River Basin Development Authority (H.J.R.B.D.A). It covers an estimated area of 3,800 sqkm^2^ (Matthes, 1990). Its maximum surface area normally occurs at the end of the rain season in September. Thereafter, the water recedes so that minimum level is reached just before the start of rains in June. The extent of flooding varies from year to year and is mainly dependent on the amount of rainfall (Benthem, 1990). Hence fish production also varies depending on the extent of the volume of water during the season. This has brought an increased fishing activity especially in those villages surrounding the dam site.

The fish specimens used for the study were obtained through catch statistics at three major landing sites of the dam, namely Marke (Site I), Dingare (Site II), and Kalgwai (Site III), respectively. Fish species were randomly sampled and examined at each site fortnightly over a period of 10 months from July 2012 to June 2013 between 6:00 a.m. and 8:00 a.m. Fish identifications as well as measurement of weight and number of fishermen were taken directly from the landing sites according to identification keys provided by Thalwar and Jhingran (1991), FAO (1992), Olaosebikan and Raji (1998) and Bankole and Mbagwn (2000).

The body weight of each specimen was determined was measured in grams (g) using compact balance (Model MP‐600A) sensitive electronic weighing scale. Catch per unit effort was determined using the formula:

$$\text{CPUE}\;=\text{No.}\;\text{of}\;\;\text{fishing}\;\text{days}\times \;\frac{\text{Total}\;\text{Weight}\;\text{of}\;\;\text{Fish}\;\text{caught}}{\text{No.}\;\text{of}\;\;\text{boats}\;\text{or}\;\text{Fishermen}}$$

Water samples from the landing sites were collected using two liter capacity sampling bottle on monthly basis. The water samples were subjected to the following analysis as soon as possible after collection as described by APHA (1998). Analysis of variance (ANOVA) was used to test for significant difference at 95% confidence limit. Data were analyzed using Minitab 14^®^ software for descriptive and summary statistics and Genstat Discovery Edition 4 for Analysis of Variance. Data were analyzed using one way analysis of variance for a completely randomized design. Some variates were analyzed using analysis of covariance where some dependent factors were found to be significantly different (*p* < .05). All significantly different means were separated using Fisher's least significant difference of means.

## 3 RESULTS

The result of catch per unit effort is as presented in Tables 1, 2, 3, 4. The number of fishers in any of the 10 months study period of the three landing sites ranged from 10 to 19, whereas the fishing hours ranged from 12 to 20. However, the average weight of fish caught per fisher per day ranged 60 kg in December to 30 kg in July, April September and October for site 1 at site 2, the average weight of fish caught per fishers per fisher is lower as it ranged from 32 to 44 kg in site 2 and 37 to 41 in site 3. (Tables 1, 2 and 3) catch per unit efforts CPUE in site 1 ranged from 18 to 70.83 kg/hr in site 3. Total weight of fish caught in all months across the three landing sites shows that in site 1 range from 18 to 70.83 kg/hr, 23.48 to 44 in site 2 and 27.79 to 42 kg/hr in site 3. Total weight of fish caught in all months across the landing sites shows that in site 1.

**Table 1:** Catch and effort data for site 1 on Kalgwai Dam

Months	No. of Fishers	Hours Fishing	Average Wt Caught/Fisher/day(kg)	No of days Fishing	Total wt/Month (kg)	Total Time (hrs)	CPUE (kg/hr)
July	17	20	30	25	12,750	500	25.50
August	15	25	30	25	11,250	625	18.00
September	18	15	30	25	13,500	375	36.00
October	16	18	30	25	12,000	450	26.67
November	14	19	50	25	17,500	475	36.84
December	12	19	60	25	18000	475	37.89
January	11	18	40	25	11,000	450	24.44
February	17	12	50	25	21,250	300	70.83
March	16	17	50	25	20,000	425	47.06
April	14	17	30	25	10,500	425	24.71
Total					147,750		

**Table 2:** Catch and effort data for site 2 on Kalgwai Dam

Months	No. of Fishers	Hours Fishing	Average Wt Caught/Fisher/day(kg)	No of days Fishing	Total wt/Month (kg)	Total Time (hrs)	CPUE (kg/hr)
July	14	19	32	25	11,200	475	23.58
August	13	20	38	25	12,350	500	24.70
September	18	18	42	25	18,900	450	42.00
October	19	19	44	25	20,900	475	44.00
November	10	15	43	25	10,750	375	28.67
December	12	16	42	25	12,600	400	31.50
January	17	18	40	25	17,000	450	37.78
February	18	18	39	25	17,550	450	39.00
March	15	18	41	25	15,375	450	34.17
April	14	19	39	25	13,650	475	28.74
Total					150,275		

**Table 3:** Catch and effort data for site 3 on Kalgwai Dam

Months	No. of Fishers	Hours Fishing	Average Wt Caught/Fisher/day(kg)	No of days Fishing	Total wt/Month (kg)	Total Time (hrs)	CPUE (kg/hr)
July	16	18	38	25	15,200	450	33.78
August	17	19	39	25	16,575	475	34.89
September	15	20	41	25	15,375	500	30.75
October	14	16	42	25	14,700	400	36.75
November	12	19	44	25	13,200	475	27.79
December	18	18	42	25	18,900	450	42.00
January	16	18	39	25	15,600	450	34.67
February	15	20	38	25	14,250	500	28.50
March	14	14	37	25	12,950	350	37.00
April	13	18	40	25	13,000	450	28.89

**Table 4:** Mean monthly catch per unit effort of fishers on Kalgwai Dam

Months	No. of fishers	Hours fishing	Average Wt. Caught/Fisher/day(kg)	No of days fishing	Total Wt./Month (kg)	Total time (hrs)	CPUE (kg/hr)
July	47	57	100	25	117,500	1,425	82.46
August	45	64	107	25	120,375	1,600	75.23
September	51	53	113	25	144,075	1,325	108.74
October	49	53	116	25	142,100	1,325	107.25
November	36	53	137	25	123,300	1,325	93.06
December	42	53	144	25	151,200	1,325	114.11
January	44	54	119	25	130,900	1,350	96.96
February	50	50	127	25	158,750	1,250	127.00
March	45	49	128	25	144,000	1,225	117.55
April	41	54	109	25	111,725	1,350	82.76
Overall	450	540	1,200	25	13,500,000	13,500	1000.00

The summary of mean monthly catch per unit effort of the fishers on the dam is presented in Table 4. The number of fishers differed significantly (*p* < .05) between month, which ranged from 41 to 51 individuals. The highest number 51 fishers were recorded in the month of September, whereas the least value 41 fishers were in April. The highest number of fishing hours of 64 hr was observed in August, whereas the lowest was obtained in March with 49 hr. The average catch per fisher per day ranged from 107 kg/day to 144 kg/day; however, the highest value of 144 kg/day was recorded in December, whereas the least value of 107 kg/day was found in August. The number of fishing days showed no significant difference (*p* > .05) with similar number of 25 days for each month. The total catch per month differed significantly (*p* < .05). The highest catch per month was recorded for February with 158,750 kg/month and the lowest value of 111,725 kg/month for April. The total fishing hours varied between 1,225 hr in March to 1,600 hr in August. The mean catch per unit effort 114.11 kg/hr was recorded for September, whereas the least value of 75.23 kg/hr was obtained for August.

The mean monthly variation in water quality parameters at the three sites is presented in Tables 5, 6 and 7. The mean monthly temperature range from 24.60 ± 0.40°C in November to 28.90 ± 0.05°C in April, 24.90 ± 0.10°C in August and September to 28.60 ± 0.10°C in March, and 24.60 ± 0.60°C in November to 28.70 ± 0.10°C in March for site 1, 2, and 3, respectively. The transparency showed no significant difference (*p* > .05) among the months, the highest value 0.31 ± 0.02 was recorded in August, whereas the least value 0.25 ± 0.01 in April for site 1, the least value of 0.26 ± 0.01 was obtained for site 2 in March and December, whereas the highest record was 0.32 ± 0.02 in July and site 3 had the highest value of 0.32 ± 0.01 in the month of August, whereas the lowest record of 0.25 ± 0.01 was observed in April. The mean monthly PH of all the sites showed no significant difference (*p* > .05). The result indicated that the highest PH values of 6.60 ± 0.10 and .65.6 ± 0.15 for site 1 and 3 in March and site 2 exhibited in the month of July and December with the value of 6.65 ± 0.15, whereas the least PH values were recorded in October 5.80 ± 0.10 for site 1 and 6.00 ± 0.10 in September for both site 2 and 3. The monthly depth of all the three sites ranged between 4.10 ± 0.10 m and 5.40 ± 0.20 m, with highest depth (5.40 ± 0.20 m) experienced July, whereas least (4.10 ± 0.10 m) in September. The result revealed that on significant difference (*p* > .05) for mean monthly dissolve oxygen at all the sites, the highest value of 6.35 ± 0.15 was recorded in November, whereas the least value of 5.55 ± 0.15.

**Table 5:** Mean monthly variation in water quality parameters at Site 1—Kalgwai Dam

Months	Temperature	Transparency	pH	Depth	DO
January	27.35 ± 0.15^bc^	0.29 ± 0.03	6.10 ± 0.30	4.55 ± 0.05^bc^	–
February	27.35 ± 0.15^bc^	0.30 ± 0.04	6.20 ± 0.10	4.50 ± 0.10^bc^	–
March	28.10 ± 0.10^ab^	0.27 ± 0.02	6.60 ± 0.10	4.50 ± 0.20^bc^	6.00 ± 0.10
April	28.90 ± 0.50^a^	0.25 ± 0.01	6.30 ± 0.10	4.55 ± 0.05^bc^	–
July	28.60 ± 0.20^a^	0.29 ± 0.01	6.15 ± 0.15	5.30 ± 0.10^a^	6.20 ± 0.60
August	25.60 ± 1.00^de^	0.31 ± 0.02	6.20 ± 0.20	5.40 ± 0.20^a^	–
September	26.35 ± 0.15 ^cd^	0.26 ± 0.02	6.05 ± 0.15	4.15 ± 0.15^c^	–
October	25.00 ± 0.00^e^	0.30 ± 0.02	5.80 ± 0.10	4.35 ± 0.15^bc^	–
November	24.60 ± 0.40^e^	0.29 ± 0.02	6.10 ± 0.10	4.45 ± 0.15^bc^	6.35 ± 0.15
December	25.10 ± 0.10^e^	0.26 ± 0.01	6.20 ± 0.30	4.60 ± 0.10^b^	–

Means in the same column with different superscripts differ significantly (*p* < .05).

**Table 6:** Mean Monthly variation in water quality parameters at Site 2—Kalgwai Dam

Months	Temperature	Transparency	PH	Depth	DO
January	27.50 ± 0.30^a^	0.28 ± 0.02	6.30 ± 0.10^abc^	4.45 ± 0.15^bc^	–
February	28.25 ± 0.15^a^	0.28 ± 0.03	6.35 ± 0.15^abc^	4.50 ± 0.10^b^	–
March	28.60 ± 0.10^a^	0.26 ± 0.01	6.50 ± 0.10^ab^	4.40 ± 0.10^bcd^	5.55 ± 0.15
April	28.50 ± 0.10^a^	0.26 ± 0.02	6.55 ± 0.15^ab^	4.50 ± 0.10^b^	–
July	26.70 ± 1.90^ab^	0.32 ± 0.02	6.65 ± 0.15^a^	4.95 ± 0.05^a^	6.05 ± 0.15
August	24.90 ± 0.10^b^	0.29 ± 0.02	6.25 ± 0.15^abc^	4.45 ± 0.15^bc^	–
September	24.90 ± 0.10^b^	0.31 ± 0.01	6.00 ± 0.10^c^	4.10 ± 0.10^d^	–
October	25.25 ± 0.25^b^	0.31 ± 0.02	6.15 ± 0.15^bc^	4.40 ± 0.10^bcd^	–
November	25.30 ± 0.70^b^	0.30 ± 0.02	6.30 ± 0.10^abc^	4.15 ± 0.05 ^cd^	6.40 ± 0.20
December	25.30 ± 0.10^b^	0.26 ± 0.01	6.65 ± 0.15^a^	4.35 ± 0.05^bcd^	–

Means in the same column with different superscripts differ significantly (*p* < .05).

**Table 7:** Mean monthly variation in water quality parameters at Site 3—Kalgwai Dam

Months	Temperature	Transparency	pH	Depth	DO
January	27.50 ± 0.10^bc^	0.27 ± 0.01^bc^	6.40 ± 0.10^abc^	4.20 ± 0.10^de^	–
February	26.40 ± 0.90^cd^	0.27 ± 0.02^bc^	6.05 ± 0.15^cd^	4.35 ± 0.15^cde^	–
March	28.70 ± 0.10^a^	0.27 ± 0.02^bc^	6.65 ± 0.15^a^	4.30 ± 0.10^cde^	5.85 ± 0.25
April	27.60 ± 0.00^ab^	0.25 ± 0.01^c^	6.50 ± 0.10^a^	4.55 ± 0.15^bcd^	–
July	24.70 ± 0.10^f^	0.30 ± 0.01^ab^	6.45 ± 0.05^ab^	4.90 ± 0.10^b^	6.40 ± 0.20
August	25.10 ± 0.10^ef^	0.32 ± 0.01^a^	6.45 ± 0.15^ab^	5.30 ± 0.10^a^	–
September	26.00 ± 0.00^de^	0.29 ± 0.01^ab^	6.00 ± 0.10^d^	4.15 ± 0.15^e^	–
October	24.85 ± 0.25^f^	0.31 ± 0.02^ab^	6.10 ± 0.10^bcd^	4.65 ± 0.15^bc^	–
November	24.60 ± 0.60^f^	0.29 ± 0.01^abc^	6.10 ± 0.10^bcd^	4.20 ± 0.10^de^	6.30 ± 0.30
December	26.05 ± 0.05^de^	0.29 ± 0.02^ab^	6.50 ± 0.10^a^	4.55 ± 0.05^bcd^	–

Means in the same column with different superscripts differ significantly (*p* < .05).

Table 8 shows the summary of the mean water quality parameters in the three sites studied on Kalgwai dam. The result indicated that no significant difference (*p* > .05) in means values of the surface temperature, transparency, depth, and dissolve oxygen, however, the PH differed significantly (*p* < .05) across all the three sites. The parameters ranged from 26.70 ± 0.36 to 26.70 ± 0.36 for temperature, transparency 0.28 ± 0.01 to 0.29 ± 0.01, PH 6.17 ± 0.06 to 6.37 ± 0.06, depth 6.37 ± 0.06 to 4.64 ± 0.09, and dissolved oxygen 6.00 ± 0.17 to 6.18 ± 0.17 across the sites.

**Table 8:** Mean water quality parameters in the three sites studied on Kalgwai Dam

Sites	Temperature	Transparency	pH	Depth	DO
Site 1	26.70 ± 0.36	0.28 ± 0.01	6.17 ± 0.06^b^	4.64 ± 0.09	6.18 ± 0.17
Site 2	26.52 ± 0.37	0.29 ± 0.01	6.37 ± 0.06^a^	4.43 ± 0.06	6.00 ± 0.17
Site 3	26.15 ± 0.32	0.28 ± 0.01	6.32 ± 0.06^ab^	4.52 ± 0.08	6.18 ± 0.16

Means in the same column with different superscripts differ significantly (*p* < .05).

## 4 DISCUSSIONS

The catch per unit effort of this study revealed the highest fish catch in February and March which was during the dry months with low rains. Similar trend was observed for the monthly total weight of fish catches. This could be attributed to the high volume of water in the dam during the rainy season and most of the species are dispersed due to the increase in surface area. This agrees with results from Jebba Lake (Halstead, 1971), Kainji Lake (Imevbore, 1975), Asa Lake (Vander‐Heide, 1982) Asejire Lake (Sendacz, Kubo, & Cestarolli, 1985), and Ikwori Lake (Offem, Ayotunde, Ikpi, Ochang, & Ada, 2011) where larger ichthyofaunal densities were observed in the dry season. Reasons for the variation were similar to those advanced in this study. However, during the wet season, it is assumed that the high level of water and subsequent flood favored reproductive activities, hence, fish species were less vulnerable to catch because of high water levels and restricted movement. This assumption agrees with the report of Willoughby and Tweddle (1978) who stated that early rainfall and subsequent rise in water level trigger spawning activities of most fish species in African water bodies.

Diversity of gear was observed used by fishermen in this study. The monthly CPUE variation might be linked to the difference in the number of fishers, man hours, and the category of fishing gears used. This is in line with the report of McClanahan, Kaunda‐Arara, and Omukoto (2010), on CPUE of closed and open‐access landing sites. The result of this study also revealed similarities in the physico‐chemical water parameters of the three sites studied. The values recorded were within the recommended ranges suggested by Boyd (1979) for the survival and development of aquatic life. The variation in the water depth recorded in per month in this study is not unconnected with rainfall in the months studied. The highest depth of 5.4 m obtained during the peak of rain in August and September is suggestive that Kalgwai Lake is relatively shallow hence, may be connected to the high fish abundance noticed in the dam. Beyond this, Alabasster and Lyod (1980) had earlier opined that high depth might result in reduction in fish growth rate as a result of reduction in food availability in the ecosystem. Hence, the low depth of the lake in this study may be an advantage translated to higher food availability.

More so, the shallow depth of this study could also be linked to the uniformity of transparency, temperature and dissolve oxygen across the landing sites. The temperature does not seem to be stratified in the Lake, thus there is free mixing of the bottom and surface layers. This vertical mixing of the two layers brings nutrients from the bottom to the surface to enhance primary productivity which accounts for high fish species richness of the Lake. Similar to the findings of this study, Famara (2002) had reported that water temperature in the Gambia estuary was not stratified with a mean difference of only 0.68°C, between the upper and lower layer of the lake. Hence, this allows the water body free vertical mixing that brings nutrients to the surface to enhance primary productivity. In general, the water quality recorded in this study is similar to the studies by Boyd (1979), Elemi (1990), Atama (2003) and the recommendations of WHO (World Health Organization) (1984) and are suitable for fish survival and development. It was concluded that the lake is not over fished and the water quality parameters are within the right range to ensure growth and survival of the fishes.

## CONFLICT OF INTEREST

None declared.
